# An integrative review on individual determinants of enrolment in National Health Insurance Scheme among older adults in Ghana

**DOI:** 10.1186/s12875-022-01797-6

**Published:** 2022-07-30

**Authors:** Anthony Kwame Morgan, Dina Adei, Williams Agyemang-Duah, Anthony Acquah Mensah

**Affiliations:** 1 Department of Planning, Kwame Nkrumah University of Science and Technology, Kumasi, Ghana; 2 Department of Geography and Planning, Queen’s University, Ontario, Canada

**Keywords:** Enabling factors, Enrolment, Need factors, NHIS, Predisposing factors, Older adults

## Abstract

**Background:**

We conducted an integrative review in an attempt to methodically and systematically understand the individual (personal factors) that influence National Health Insurance Scheme [NHIS] enrolment among older adults aged 50 years and above. The study was premised on evidence pointing to a state of little or no change in the enrolment rates [especially among older adults], which contrasts with the initial euphoria that greeted the launch of the scheme - which culminated in high enrolment rates.

**Methods:**

The integrative literature review was conducted to synthesise the available evidence on individual determinants of NHIS among older adults. The methodological approach of the integrative literature review follows a five-stage interdependent and interconnected procedure of problem identification, literature search, data evaluation, data analysis and results presentation. Studies that met the inclusion criteria were peer-reviewed articles published in the English Language, from January 2010 to July 2020 and have Ghana as its setting or study area. The Andersen's Behavioural Model was used to categorize the predictor variables.

**Results:**

Predisposing factors [gender, age, level of education and marital status], enabling factors [income] and need factors [health conditions or health attributes of older adults] were identified as individual predictors of NHIS enrolment among older adults. The findings support argument of Andersen's Behavioural Model [where predisposing, enabling and need factors are considered as individual determinants of health behaviour].

**Conclusions:**

The findings call for policy reforms that take into account the aforementioned individual predictors of NHIS enrolment, especially among the aged.

## Background

Around the globe, developed countries like Germany, Austria, Japan, Belgium, South Korea, and developing countries like Israel have somehow achieved universal health coverage [UHC] through social health insurance [SHI] [1] with some countries reaching this milestone over a shorter period as compared to others. Several low and middle-income countries [LMICs] are therefore experimenting with different health financing models, for instance, social health insurance and UHC schemes [2–5]. These efforts signify attempts to undo healthcare utilization barriers and financing gaps that hinder access to preventive and curative medical services among vulnerable populations, particularly the older adults . Health insurance through pooled funding is seen as an income redistributive approach, that results in a better access to health services among vulnerable groups by equalising the potential to pay for services [6, 7]. Its importance is seen in providing a certain level-ground in healthcare accessibility and utilisation.

Ghana's journey to achieving UHC commenced in the 1990s with the operationalisation of community-based health insurance (CBHI) schemes within some districts of the country. Non-governmental organisations (NGOs) were at the front of the CBHI schemes with assistance from the international community [8, 9]. Evaluation of the CBHI schemes shows they covered less than 1% of the Ghanaian population with limited benefits, primarily inpatient services [9]. As reported by Waddington & Enyimayew [10], the out-of-pocket [OOP] payment for healthcare services improved quality of care and supply of drugs but vulnerable groups were denied access, due to inability to pay. The situation created gaps in financial access to healthcare services, thereby resulting in inequity and deteriorated health outcomes, and in some instances avoidable deaths [10–12]. This unsustainable healthcare financing has been criticised in academic, policy and media landscape, with calls for reform “flooding” all facets of the Ghanaian national life.

The Government of Ghana [GoG] introduced the National Health Insurance Scheme [NHIS] in 2003 through the enactment of the National Health Insurance Act (Act 650 of 2003) and Legislative Instrument (LI) 1809 [4]. The scheme is a financial risk protection or buffer to all Ghanaians and legally resident non-Ghanaians against the need to pay healthcare user fees at the point of service use [1]. The National Health Insurance Act of 2003, Act 650 stipulates the detailed diseases and healthcare services covered and not covered under the scheme [13]. Currently, the premium set by the National Health Insurance Authority [NHIA], the administrator of the scheme, is a minimum of GH₵6.00 [$0.80] and a maximum of GH₵42.00 [$4.60], whiles the card processing fee is set at GH₵6.00. People below 18 years pay an enrolment fee of GH₵ 6.00 [$0.80], active social security and national insurance trust [SSNIT] contributors GH₵ 6.00 [$0.80] and all other adults (18 – 69 years) GH₵ 28.00 [3.73]. Exemptions are however, provided for vulnerable groups: children below 18 years, older adults (70 years and above), indigents, pregnant women and mentally challenged persons.

As a SHI scheme, Ghana’s NHIS is a voluntary health protection scheme that provides financial buffer, against the payment of healthcare utilization fees at points of use, contingent on enrolment and continuous membership renewal. Enrolment peaked during the early years of execution and stagnated around 40% of the population between 2011 and 2015 [11]. Year-on year enrolment rates as percentage of the total population reveals that between 2015 and 2017, enrolment in the scheme stagnated around 60% of the population. Implying that the awe-inspiring initial popularity of the NHIS has not transformed into high enrolment and regular membership renewal in recent years [4, 11, 14]. Evidence-based tailored policies to reduce membership enrolment delays, improve positive perceptions and awareness of the NHIS, and address financial barriers to enrolment among some groups can be positive precursors to improving trust and enrolment and addressing broad equity concerns about the NHIS.

Empirical studies conducted since the implementation of the health insurance scheme in 2003 so far presents inconsistent and differing views on the determinants of enrolment in the scheme [15–21]. Individual factors [sociodemographic and need factors] [6, 16, 18, 22–24], scheme and health system factors [inadequate information about insurance scheme and perceived poor quality of service] [7, 22, 25–27] are the reported determinants of enrolment. The results show that determinants of enrolment in the NHIS are multi-faceted.

This paper presents an integrative review on the individual predictors of health insurance enrolment among older adults in Ghana by employing the Andersen's Behavioural Framework [where predisposing factors affect attitude towards insurance; enabling factors facilitate or inhibit an individual’s effort to enrol; and perceived health status represents need factors] [20, 28]. Older adults [in this context denotes persons 50 years or older] [29, 30] who are more prone to chronic diseases, which lead to reduced capacities to earn income [31–34], have higher dependency rates and susceptibility to facing financial barriers to healthcare use [35–38]. While an argument could be made that a significant majority of older people will fall within the range of exempt groups (70 years and above and indigents, in addition to SSNIT pensioners), socio-demographic determinants of health insurance enrolment such as age, education, gender and marital status are often used to explain why some individuals with high wealth index will still do not enrol in health insurance schemes. In light of this, the paper provides a grounded framework to guide policy and practice on individual determinants of enrolment in the NHIS among older adults in Ghana. This is in line with lessening barriers to enrolment in the scheme, since subscription and continuous renewal of NHIS membership guarantees free healthcare use for some predefined health problems, serving as a sustainable healthcare financing [particularly for the poor and vulnerable populations] and a means towards the realization of Sustainable Development Goal 3.

## Methods

### Methodology

The integrative literature review [a methodology that allows for the inclusion of non-experimental and experimental research along with empirical and theoretical literature to provide the extensive form of review method] [39, 40] was conducted to synthesise the available evidence on the individual determinants [predisposing, enabling and need factors] of NHIS enrolment among older adults in Ghana. An integrative literature review provides an abridged outlook on erstwhile theoretical and/or empirical research [41–43], which enables a researcher(s) to have a complete understanding of a particular problem. The approach was seen as the most appropriate to gain a complete understanding of the phenomenon with the purpose of using the conclusions as guiding principle to improve the implementation of the National Health Insurance Policy [NHIP]. The methodological approach of the integrative literature review follows a five-stage interdependent and interconnected procedure [40, 43, 44]. These include (1) problem identification; (2) literature search; 3) data evaluation; (4) data analysis; and (5) presentation of results.

Andersen's Behavioural Model which aims to understand how and why people use healthcare services, assess inequality in access to health services, and aid in the formulation of policies that will allow for equitable access to healthcare was used as the philosophical underpinning of the study. To predict or explain one's enrolment in SHI, the model focuses on an individual's predisposition to enrol, enabling factors that facilitate or inhibit enrolment, and one's perceived or influenced need for care. With the use of this model, one is able to assess individual measures of enrolment (e.g., age, gender, marital status, educational level, income, health condition) impacting enrolment in the NHIS in Ghana.

### Inclusion criteria

The focus of the integrative review centred on individual determinants of NHIS enrolment among older adults in Ghana. This included core areas such as predisposing factors [age, gender, level of education, family size, occupation, marital status, peer influence and health principles and attitudes, among others], enabling factors [(income, place of residence, knowledge of insurance] and need factors [health conditions or health attributes that increase an individual’s demand for a health commodity]. Specifically, the included papers addressed individual determinants of enrolment in the scheme with empirical studies on health protection schemes in Ghana – with focus on older adults. Studies that met the inclusion criteria were peer-reviewed articles published in the English Language, from January 2010 to July 2020 and have Ghana as its setting or study area - since research on the predictors of enrolment in the NHIS that focuses on older adults or have a disagregated data on older adults rose to prominence from 2010.

### Exclusion criteria

The articles excluded were studies that discusses other determinants [scheme-related factors and health system factors], rather than individual determinants of enrolment in the scheme. Other general exclusion criteria were conference abstracts, book chapters, papers that present opinion, editorials, commentaries and reviews. The exclusion of reviews from the eligible papers was hinged on its greater propensity of distorting reality [45, 46]. The exclusion of reviews of any form from the eligible papers was also premised on the basis that reviews are not empirical studies – but secondary studies that rely on evidence from existing articles. In this study, we set out to review only empirical papers as such, the inclusion of reviews of any form will have constituted an anomaly. Additionally, studies conducted before 2010 and in any other parts of the world other than Ghana were also excluded – since research on the predictors of enrolment in the NHIS shot into the limelight from 2010 onwards. The exclusion of papers published prior to 2010 is premised on fact that these studies were largely on financing of the scheme, effects of enrolment on service utilization for specific ailments, challenges confronting the scheme, and factors contributing to low enrolment in the scheme, without disaggregated evidence for older adults on determinants of enrolment in the health protection scheme. These studies were therefore considered inconsequential to the attainment of the study’s objective.

### Search strategy and selection procedure

Literature search is aided by the formulation of research questions, with the goal to retrieve relevant papers or articles [43, 47]. The review question was conceptualised using the PICO (T) approach [population, intervention, comparative intervention, outcome and time] [47, 48]. PICOT mirrors the essentials of the research question and is an upheld pedagogical model [43, 49]. The PICOT components were (P) older adults, (I) NHIP or the scheme, (C) no comparisons were made, (O) enrolment in the NHIS and (T) January 2010 to July 2020 [49].

The search for published articles was conducted in seven electronic databases, namely PubMed, Web of Science, Scopus, PsycINFO, Science Direct, Ovid, and Sage. The search strategy was developed based on the suggested guidelines by the Joanna Briggs Institute [JBI] [50, 51]. Precisely, a three-step search strategy was adopted to aid the search for articles (refer to Table 1). An initial restricted search was conducted in PubMed and Web of Science. The text words within the title and abstract and of the index terms from the initial search results were analysed [51]. A second search employing all the index terms and the identified keywords were then repeated across the five remaining databases (refer to Table 1). Finally, the reference lists of all eligible [but electronically missed] studies were manually searched or hand searched [50–55].

**Table 1:** Search strategy and selection procedure

Stage	Database	Search Terms and Keywords
One	(Initial search in PubMed and Wiley Web of Science)	ALL (“enrol”*) AND (“health insurance” OR “NHIS”) AND (“individual facilitators” OR “individual enablers”) (“older adults” OR older people OR “adults”)
Two	(search across, Scopus, PsycINFO, Science Direct, Ovid, and Sage)	ALL (“enrol”* OR “register”* OR “join”) AND (“health insurance” OR “NHIS” OR “health protection”) AND (“predisposing” OR “need” OR “enabling” OR “motivations”) AND (“older adults” OR older people OR “adults” OR “older folks” OR “older individuals”) (“enrol”* OR “register”* OR “join” OR “subscribe”) AND (“health insurance” OR “NHIS” OR “health protection” OR “health cover”) AND (“personal factors” OR “enablers” OR “motivators” OR “motivations” OR “drivers” OR “stimulus”) AND (“older adults” OR older people OR “adults” OR “older folks” OR “older individuals” OR “elderly”)
Three		Hand searching of the reference lists

**Fig. 1:**
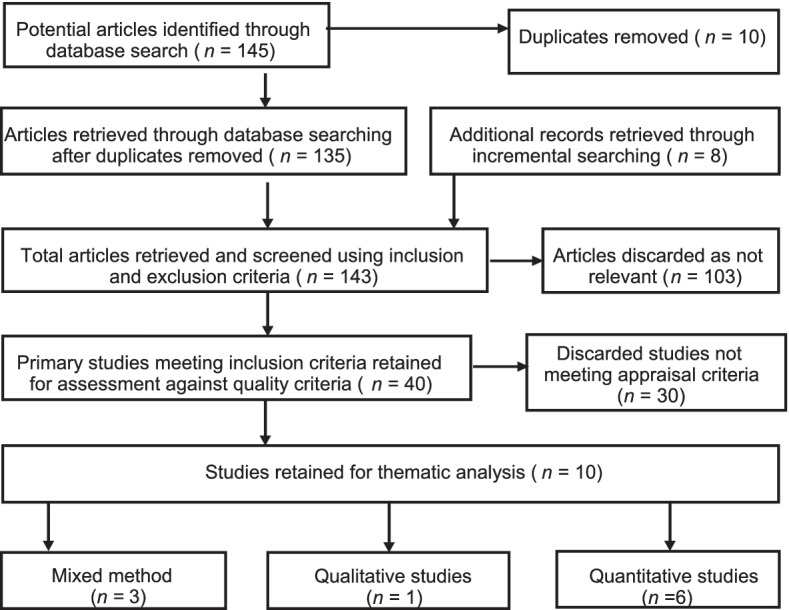
Flow Chart of Studies Included in the Review based on PRISMA

The selection of eligible articles adhered to the Preferred Reporting Items for Systematic Reviews and Meta-Analyses [PRISMA] [50, 56] (see Fig. 1). Five researchers [the four authors and an independent researcher] critically and independently appraised the titles of articles that were retrieved and approved those meeting the selection criteria. The role of the independent reviewer was to serve as auditor, who double-checks the accuracy and usefulness of the entire approach, to attaining the study’s objective, and limiting bias. The titles and abstracts of the downloaded papers were reviewed and agreement was reached on those needing full-text screening. Full screening was done in accordance with the inclusion and exclusion criteria.

### Data management and extraction

Standards for the appraisal of the validity and significance of scientific literature are not fixed or inert [57, 58]. However, a structured method to critical evaluation could hypothetically improve the quality of the process, and checklist can be expedient to screen out low quality from high quality research [57]. The guide to a critical appraisal of literature by Young & Solomon [57] was used to appraise the selected articles. The data extraction form was categorised into sub-sections, such as study details (citation, year of publication and author), objective of the paper, and the primary subject area of the paper (predisposing, enabling or need factors). The four researchers [authors] critically appraised the ten studies and retained all for analysis.

The methodological quality of the included papers were evaluated in the subsequent groupings: suitability of the research objective, data collection, and methodological adequacy, and study design, recruitment of participant, data analysis, and results presentation. The quality of the included studies classified using a grading system. A quality grade stretching from 50% (low quality), 51–75% (average quality), and 76–100% (high quality) were developed to guarantee that the included designs are appropriate for the study objectives. The quality evaluation also aided in reporting bias risk, reviewing the quality of evidence, sufficiency and methodology, research design, participant enlistment data collecting, analysis of data, and the presentation of outcomes. From the results presented in Table 2, the study of Van der Wielen et al. [6] ranked first with a score of 88 (high quality), Van der Wielen et al. [7] and Salari et al. [15] in second with a score of 87 (high quality) and Van der Wielen et al. [25] in third with a score of 86 (high quality). The study of Baozhen et al. [56] ranked lowest in terms of methodological quality, with a score of 50 (low quality). Overall, these studies contained relevant information that addressed the study’s objective.

**Table 2:** The methodological quality of the included papers

SN	Author(s)	Year	Score	Range
1	Ayitey et al.	2013	69	Average Quality
2	Alatinga & Williams	2015	65	Average Quality
3	Baozhen et al.	2019	50	Low Quality
4	Duku et al.	2015	74	Average Quality
5	Kotoh et al.	2018	65	Average Quality
6	Parmar et al.	2014	77	High Quality
7	Salari et al.	2019	87	High Quality
8	Van der Wielen et al.	2018a	88	High Quality
9	Van der Wielen et al.	2018b	87	High Quality
10	Van der Wielen et al.	2018c	86	High Quality

Inter-rater reliability, which affords a comparison of the measure of the same event by more than one observer [59] was used to establish the quality of the tool [60]. The Spearman Correlation Coefficient was used to determine inter-rater reliability. A correlation was found between reviewer 1 and 2 (p <.0000), between reviewer 1 and reviewer 3 (p<0.006) and between reviewer 1 and 4 (p<0.041). A correlation was established between 2 and 3 (p<0.005), between reviewer 2 and 4 (p<0.044) and between reviewer 3 and 4 (p<0.036) at 5% significant level. The strongest correlation exists between reviewer 1 and 2 whiles the weakest correlations exist between reviewer 1 and 3. Refer to Table 3 for more details.

**Table 3:** The inter-rater reliability between the reviewers

Spearman Correlation Coefficients, N=11 Prob > [r] under H0: Rho = 0				
	Reviewer 1	Reviewer 2	Reviewer 3	Reviewer 4
Reviewer 1		0.940^a^	0.765^a^	0.816^a^
		0.000	0.006	0.041
Reviewer 2	0.940^a^		0.774^a^	0.811^a^
	0.000		0.005	0.044
Reviewer 3	0.765^a^	0.774^a^		0.771^a^
	0.006	0.005		0.036
	0.816^a^	0.811^a^	0.771^a^	
	0.041	0.044	0.036	

^a^Correlation is significant at the 0.05 level

### Data synthesis

The thematic approach was employed to synthesise evidence from the extracted data [61, 62]. The thematic analysis involved several stages which comprise the identification of patterns, seeing plausibleness, clustering, making comparisons and subsuming particulars, noting relationships between variability and finding intervening factors [63]. See Table 4 for details. The thematic review was done in line with the Andersen's Behavioural Model where predisposing, need for care and enabling factors became a band of themes under which the data were grouped.

**Table 4:** Description of the reviewed articles

SN	Author(s)	Year	Region(s)	Objective	Sample size
1	Ayitey et al.	2013	All 10 regions in Ghana before the regional redemarcation in 2019.	The study investigates the determinants of older adults’ decision to enrol in social health protection schemes in Ghana.	8,687 households
2	Alatinga & Williams	2015	Kassena-Nankana District, Upper East region	Investigates the determinants of households’ decision to enrol in social health protection schemes in Ghana.	417 household heads
3	Baozhen et al.	2019	Ashanti Region	The study investigated the factors influencing the patronage of Ghana’s NHIS.	60 households
4	Duku et al.	2015	Western and the Greater Accra Regions in Ghana	The extent to which the NHIS exemption policy for older adults influences or predicts enrolment in the scheme was investigated in this study.	4214 individuals aged 18 years and above of which 933 are older adults aged 50 years and above).
5	Kotoh et al.	2018	Central and Eastern Regions of Ghana	Factors that influence the enrolment and retention in Ghana’s NHIS.	3000 households
6	Parmar et al.	2014	Central, Eastern, Ashanti, Brong-Ahafo and Northern regions	The study explored how the social, political, economic and cultural (SPEC) dimensions of social exclusion influence the enrolment of older adults in social health protection programs.	435 older adults
7	Salari et al.	2019	All 10 regions in Ghana before the regional redemarcation in 2019.	The study investigated the predictors of NHIS enrolment among Ghanaians, using disaggregated data from three national household surveys (Demographic and Health Survey [DHS], Multiple Indicators Cluster Survey [MICS], and Ghana Living Standards Survey [GLSS]	DHS (12,831 households) MICS (12,150 households) GLSS (18,000 households)
8	Van der Wielen et al.	2018a	All 10 regions in Ghana before the regional redemarcation in 2019.	Determinants of national health insurance enrolment in Ghana across the life course using data from the 2007-2008 Study on Global Ageing and Adult Health (SAGE) and the 2012-2013 Ghana Living Standards Survey (GLSS) was the study’s focus.	16,772 households from GLSS 5,110 individuals from the SAGE survey
9	Van der Wielen et al.	2018b	All 10 regions in Ghana before the regional redemarcation in 2019.	The objective of the paper was to examine the community, household and individual level determinants of NHIS enrolment among older adults aged 50–69 and 70 plus, using data from the 2012-2013 Ghana Living Standards Survey (GLSS).	16,772 households from GLSS
10	Van der Wielen et al.	2018c	All 10 regions in Ghana before the regional redemarcation in 2019.	The study investigated whether insurance enrolment increase healthcare utilisation among rural dwelling older adults, using data from the 2012-2013 Ghana Living Standards Survey (GLSS).	16,772 households from GLSS

## Results

### Description of the reviewed articles

The ten articles that met the inclusion criteria were primary studies conducted on enrolment in the NHIS among older adults [explicitly or contained disaggregated information on older adults] and published in the English language (see Table 4 for details). Six of these studies employed a quantitative research approach; three of these studies employed a mixed methods or the pragmatic research approach; whereas one of these studies employed a qualitative research approach. The papers addressed the different individual or personal determinants [predisposing, enabling and need factors] of NHIS enrolment among older adults in Ghana (see Table 5 for details).

**Table 5:** The key emerging themes

Themes	Sub-themes	N^a^	Papers
Predisposing factors	Gender	7	[7, 16, 25, 64–67]
	Age	5	[7, 16, 25, 65, 67]
	Level of education	8	[6, 7, 15, 25, 64–67]
	Marital status	9	[6, 7, 15, 16, 25, 64–67]
Enabling factor[s]	Income	9	[6, 7, 15, 16, 25, 64–67]
Need factors	Health conditions or health attributes	6	[6, 25, 15, 16, 61, 63]

*N*^*a*^ Number of papers

### Predisposing factors

The predisposing factors affect attitudes towards a health activity. In this case, a predisposing factor is defined as elements of personhood that affects the individual’s attitude and behaviour towards enrolment in the health protection scheme. These [predisposing] factors are gender, age, level of education, and marital status of older adults.

#### Gender

Seven studies highlighted gender as a determinant of NHIS enrolment among older adults in Ghana [7, 16, 25, 64–67]. All seven but one study associated higher enrolment rates to the female gender. These studies [7, 16, 25, 64, 66, 67] reveal that females or female-headed households had a higher odd of enrolling in the NHIS. This may be a consequence of higher prospects of healthcare usage among women as compared to men. Contrastingly, Alatinga & Williams [65] established that male-headed households were more likely to enrol in the scheme than female-headed household; with the feminisation of poverty being the underlying reason.

#### Age

Age was another predisposing factor identified as a determinant of enrolment in the NHIS by older adults. Specifically, five articles established the association between age and NHIS enrolment [7, 16, 25, 65, 67]. All studies discovered that as age cohort of older adult’s increases, the rate of enrolment increases. For instance, older adults aged 70 years or above were more likely to enrol in the scheme than their counterparts aged between 50 to 59 years or those aged between 60 to 69 years [7, 16, 25, 67]. Alatinga and Williams [65] also made the point that older adults aged 60 years and above were twice as likely to enrol in the NHIS as compared to older adults who are less than 60 years. Higher healthcare needs of older adults, alongside the exemption policy-that grants free enrolment to a certain category of the Ghanaian population of which older adults form part were put forward as reasons for higher enrolment among older adults aged 70 years or above.

#### Level of education

Eight studies found an association between level of education of older adults and enrolment in the scheme [6, 7, 15, 25, 64–67]. Older adults with higher education have a higher tendency to enrol in the scheme [6, 7, 15, 25, 64–67]. The influence of education on enrolment decision is underscored by its ability to increase one's knowledge about the benefits of health insurance. However, higher education is also associated with lower risk [both actual and perceived] and thereby reducing the propensity to buy health insurance. Parmar et al. [66], however found older adults with higher education to have a lower tendency to enrol in the scheme in comparison to older persons with no formal education. Salari et al. [15] likewise found no association between level of education and enrolling in the scheme among older adults in the Demographic and Health Survey (DHS) when the authors compared enrolment among older adults in three national household surveys (Demographic and Health Survey, Multiple Indicator Cluster Survey, and Ghana Living Standards Survey). A situation attributed to increased awareness of the intervention and its possible benefits.

#### Marital status

Marital status as a determinant of NHIS enrolment was found in nine of the articles reviewed [6, 7, 15, 16, 25, 64–67]. Married older adults were more likely to enrol in the NHIS compared to those never married, separated or widowed [6, 7, 15, 16, 25, 64–67]. The perceived need to reduce family healthcare expenditure was put forward as the primary reason for higher enrolment rates among married older adults than older adults who have never married, divorced, widowed or are cohabiting. On the contrary, Duku et al. [16] discovered that older adults who have never married and are cohabiting were 1.23 and 1.11 times more likely to enrol in the scheme as compared to older adults who were married.

#### Enabling factors

The enabling factors [income, place of residence, knowledge of insurance] relate to factors that facilitate or promote an individual’s effort to enrol in the scheme. They are positive enforcers that promote the ability of an older adult to register with the scheme. The focus of enabling factors in this analysis was on income, since the papers did not report on place of residence or knowledge of insurance as determinants of enrolment decision. Nine studies identified income or socioeconomic status as the major enabling determinant of enrolment in the scheme [6, 7, 15, 16, 25, 64–67]. For instance, older adults belonging to richer households (Q3 and Q4) were 2.6 and 4 times more likely to enrol in the NHIS as compared to the poorest 25% households [66]. Also, older adults who earn a monthly income of between $4 and $27 were 0.47 times less likely to enrol in the NHIS. In other words, the chances or probability of these older adults actually enrolling in the NHIS is 0.47 times less as compared to older adults that earn between $32 and $46 [65]. The review also revealed that the rich and more affluent were more likely to enrol in the scheme as compared to the poor [6, 7, 15, 16, 25, 64]. This stems from the fact that premiums serve as a barrier to the enrolment of poorer households in health protection schemes. The results show that wealth status is a significant determinant of NHIS enrolment as well-to-do older adults have a higher odd of enrolling in the scheme. This pro-rich predisposition in the NHIS enrolment across some of the studies illustrates that the scheme has failed to meet the needs of the poorest to a certain degree [6, 25].

### Need factors

Need factors as determinants of health behaviour denote specific health conditions or health attributes that increase an individual’s demand for a health commodity. In this case, a need factor constitutes any health-related attribute that will make an individual more likely to enrol in the health insurance scheme. In all, six papers reported on need factors as determinants of health insurance enrolment [6, 15, 16, 25, 64, 66]. Specifically, being hospitalised in the last 12 months preceding the data collection increased the odds of enrolling in the scheme by four folds, according to Parmar et al. [66] while Salari et al. [15] also found that recent use of health services [two weeks prior to the gathering of the Ghana Living Standards Survey data and six months prior to gathering the Demographic Health Survey data] was associated with an increased rate of enrolment in the scheme. Thus frequent use of healthcare services due to underlying health or medical condition (chronic condition) increases the odds of enrolling in the scheme; a finding which was also reported by Parmar et al. [66]. Older adults with good self-rated health were less likely to enrol in the scheme due to the low or reduced perceived need of the scheme [16]. Duku [64] also found that older adults with poor self-rated health were more likely to enrol in the NHIS than those with fair or good health [although the finding was statistically insignificant]. Additionally, older people with disability were significantly less likely to enrol in the NHIS [6, 25]. This may point out that access to either services or the registration process is affecting enrolment amongst this group [people with disabilities], thus deepening their predisposing vulnerability.

## Discussion

### Predisposing factors

Gender has been an age-long determinant of health behaviour, as some health behaviours are more associated with a particular gender. Most of the studies reported females to be significantly more likely to enrol in the health protection scheme [NHIS] as compared to males. That is to say that being a female was associated with a higher predisposition to enrol in the health protection scheme. This confirms evidence from previous studies [20, 68, 69] where females were reported to have higher NHIS enrolment rates than males. Dixon et al. [70] ascribed this to the tendency of women taking responsibility for the health and wellbeing of the family - and are consequently more likely to be aware of the benefits provided by health insurance coverage. While on the average, women are less well-off than men in Ghana, the situation is rapidly changing [particularly in the light of increasing economic empowerment of women], making the likelihood of the former enrolling in the scheme higher than that of the latter, in the majority of the papers that reported on gender and NHIS enrolment.

The motivational component of the NHIS [the scheme serving as a financial buffer against the payment of healthcare utilization cost] might have played a significant role. Despite the financial commitment required, enrolling could provide a handy means of financing healthcare in times of need, offering the prospects for women [mostly vulnerable], to invest in the NHIS for future healthcare utilization security. Digging deeper, the higher predisposing of females to use formal healthcare services could also explain their higher propensity to enrol in the scheme, compared with their male counterparts. Thus applying basic health economics, offsetting the cost associated with frequent service use could motivate the payment of a one-time premium to guarantee healthcare use, at least for a year. Efforts aimed at promoting women empowerment must be strengthened, since it will improve older women’s enrolment in the scheme and an overall positive health outcome, all things being equal.

On the contrary, one of the papers [65] found males to have a higher rate of enrolment than females; an inclination towards evidence from previous studies [71, 72]. The feminisation of poverty in Africa [a situation in which female-headed households lack access to, and control over resources, such as land] is often attributed to the lower likelihood of females and female-headed households to patronise health protection policies. Rightly so, the study of Alatinga & Williams [65], conducted in the Kassena-Nankana District of Ghana’s Upper East Region, a region known for male dominance and poorer socioeconomic status of women. This could explain their lower predisposition to enrol in the scheme, compared to males. Policies that empower women [economically, socially and politically] must be pursued aggressively alongside factoring gender issues into the health protection scheme.

The evidence from the articles established that older adults [more advanced in age] were more likely to enrol in the scheme as compared to younger adults. Specifically, those above the age 70 were more likely to enrol as compared to those between 50 to 59 years and 60 to 69 years. A situation often attributed to the higher healthcare needs of older adults, alongside the exemption policy-that grants free enrolment to a certain category of the Ghanaian population of which older adults [70 years and above] form part. This result corroborates evidence established in previous studies [73–78] that older adults have a higher tendency to enrol in health protection schemes.

Furthermore, an individual’s level of education was established in the study as an important determinant of enrolment among the older adults. Specifically, the majority of the articles reviewed reported positive association between higher educational attainment and enrolment in the scheme. Past studies suggest higher education leads to a greater appreciation of health insurance schemes and a higher probability to enrol [20, 68, 69, 79, 80]. Higher education opens the prospect for older people to know more about the benefits of SHI schemes and the need to enrol, leading to higher enrolment rates in SHI schemes among literate older adults. That said, Kimani et al. [81] argued that having health insurance coverage is concomitant with an individual’s educational status, although higher education could weaken the need factor [though higher awareness and abstinence from behaviours that could predispose an individual to require frequent utilisation of health services].

The review further established a link between the marital status of older adults and enrolment in the NHIS in Ghana. Precisely, married older adults were found to be more likely to enrol in the health insurance compared to single, widowed or divorced older adults. A large body of literature on socio-demographic determinants of enrolment in health protection schemes portrays that higher enrolment is associated with individuals who are married [18, 68, 82–84]. The higher likelihood of married older adults to enrol in health protection scheme is premised on the need to offset household expenditure by relying on the scheme to cater for health expenditure. On the contrary, evidence also abounds that the marital status of individuals does not predict rates of enrolment in health protection schemes [23, 24].

### Enabling factors

Enabling factors promote or empower an individual to undertake a health activity. In our opinion, enabling factor [income in this instance] facilitate or inhibit an individual’s effort to enrol in the scheme. Older adults from poorer socio-economic backgrounds had a lower propensity to enrol in the scheme. This stems from their inability to afford the enrolment fee. The finding is consistent with studies that found considerable evidence of low enrolment for the most economically vulnerable individuals in social health protection schemes that are precisely targeted towards the poor in LMICs [20, 21, 76, 85]. Similar studies in Cameroon, Kenya and Senegal also highlight income as an important enabling factor that determines enrolment in health protection schemes [81, 86, 87]. Within the health economics literature in sub-Sahara Africa, poverty has been given excessive weight as the main determinant health insurance coverage [4, 22, 26, 27, 88, 89]. Due to the lower enrolment of people from poorer socioeconomic backgrounds in the scheme, some of the papers described the scheme as a near pro-rich bias health protection scheme that requires considerable modifications to meet the needs of the “hardcore poor.” Income and socioeconomic status must be critically factored into the formulation of health insurance policy in Ghana.

### Need factors

The study largely established that older people with underlying health conditions have a higher tendency to enrol in the scheme compared to older adults who have no underlying health conditions. This supports the argument that the propensity to undertake a particular health behaviour is hinged to some extent on the belief that one is prone to the condition and that the behaviour could prevent or undo the occurrence [90, 91]. For this reason, older adults who believe they have a higher tendency of falling ill and that insuring against such uncertainty would help to prevent the occurrence of the financial hindrance to healthcare use act on that need. As a result, that need attribute increases their demand for health insurance. Further, the finding confirms earlier studies where individuals with poorer health status are more likely to enrol in the NHIS in the USA [92], Ghana [93], as well as in other LMICs [94]. This implies that health risks and vulnerabilities to ill-health need to be appropriately communicated to engender the need to register in the scheme among older adults [especially considering their much higher demand for health services]. The study also found older persons with disabilities were less likely to enrol. This could mean access to either services or the registration process is affecting enrolment among this group [older persons with disabilities], thus deepening their predisposing vulnerability. Other factors beyond financial challenge [which is somehow addressed by the exemption policy] such as long travel time to registration centres and time constraint in addition to transportation barriers could be producing the lower enrolment rate among older adults with disabilities. Studies need to explore the underlying causes such that solutions can be provided to improve enrolment among this group.

#### Implications for policy, practice and research

This integrative review on individual or personal determinants of NHIS enrolment among older adults in Ghana discovered gender, age, level of education and marital status, income and perceived health status to be associated with NHIS enrolment. These factors must be considered in devising strategies to increase enrolment rates in the NHIS among older adults. It presupposes that a holistic approach should be adopted when considering the individual determinants of enrolment in the scheme. Policy reforms should reflect the various individual dimensions to generate the needed impact. Further, a comparative study on individual determinants of HNIS enrolment among older adults between rural and urban areas in Ghana is recommended. The recommendation further suggests a comparative study between various regions on the individual determinants of NHIS enrolment among older adults to guide localised interventions and policies. Finally, future research should explore predisposing factors such as family size, occupation of older adults, and peer influence in addition to health principles and enabling factors like place of residence and knowledge of insurance that were not captured in this review.

#### Limitations of the study

The study has some limitations despite the notable contribution it makes to literature on determinants of enrolment in health protection schemes among older adults. First, the review was silent on predisposing factors such as family size, occupation of older adults, and peer influence in addition to health principles among other attitudes. In addition to gender, age, level of education and marital status, these other predisposing factors possess significant influence over the decision of individuals [older adults] to undertake a health initiative. As such, their exclusion from the analysis could impact the appreciation of the subject matter. Second, the approximation of income as the only enabling factor in the paper limit the roles of place of residence and knowledge of insurance as potential determinants of enrolment decisions among older adults. Future studies should explore the impact of these enabling factors on enrolment decisions of older adults. Third, we focused on a single country with unique conditions such as cultures and norms. This limits the generalisability of the findings to other countries. Finally, a major limitation of this review is the limited number of articles included in the study. Only 10 papers were analyzed in the end, and this presents a situation of limited data. This led to the inability to perform a meta-synthesis.

## Conclusion

The search for a sustainable healthcare financing module [especially for the poor and vulnerable populations] led to the implementation of the NHIP under the tutelage of NHIA in Ghana. Under the policy, citizens are to access healthcare for some selected ailments free of charge - pending enrolment in the scheme and a continuous yearly renewal of membership. From the initial euphoria that greeted the launch of the scheme - as culminated in high enrolment and renewal rates, recent evidence points to a general plateau in the enrolment rates. This has birthed a plethora of research into the determinants of enrolment in the scheme - especially among vulnerable populations of which older adults form part. The cumulative nature of science requires the need to find, evaluate and synthesise evidence [an endeavour which lends much credence to evidence-based practices] in health policy and planning. This paper bridges the void (lacuna) that absence of synthesised literature creates and offer prospects to improve enrolment rate among older adults - a known population with higher healthcare demands [needs] - but [often] with weak financial capabilities. The study found predisposing factors [gender, age, level of education and marital status], enabling factors [(income] and need factors [health conditions or health attributes] to be associated with NHIS enrolment among older adults. The findings supports the tenets of theory adopted [predisposing, enabling and need factors as determinants of health behaviour]. The above-highlighted findings of the study call for policy reforms that take into account the said individual predictors of NHIS enrolment.

## Data Availability

No datasets were generated or analyzed during the current study.

## Abbreviation

CBHI: Community-Based Health Insurance
DHS: Demographic Health Survey
GLSS: Ghana Living Standards Survey
LMICs: Low and Middle-Income Countries
NHIA: National Health Insurance Authority
NHIP: National Health Insurance Policy
NHIS: National Health Insurance Scheme
NGO: Non-governmental organisations
OOP: Out-Of-Pocket
SDGs: Sustainable Development Goals
SHI: Social Health Insurance
SSNIT: Social Security and National Insurance Trust
UHC: Universal Health Coverage
